# Anatomy of an extensively drug-resistant *Klebsiella pneumoniae* outbreak in Tuscany, Italy

**DOI:** 10.1073/pnas.2110227118

**Published:** 2021-11-24

**Authors:** Melissa J. Martin, Brendan W. Corey, Filomena Sannio, Lindsey R. Hall, Ulrike MacDonald, Brendan T. Jones, Emma G. Mills, Casey Harless, Jason Stam, Rosslyn Maybank, Yoon Kwak, Katharina Schaufler, Karsten Becker, Nils-Olaf Hübner, Stefania Cresti, Giacinta Tordini, Marcello Valassina, Maria Grazia Cusi, Jason W. Bennett, Thomas A. Russo, Patrick T. McGann, Francois Lebreton, Jean-Denis Docquier

**Affiliations:** ^a^Multidrug-Resistant Organism Repository and Surveillance Network, Walter Reed Army Institute of Research, Silver Spring, MD 20910;; ^b^Dipartimento di Biotecnologie Mediche, University of Siena I-53100 Siena, Italy;; ^c^Veterans Administration Western New York Healthcare System, University at Buffalo, State University of New York, Buffalo, NY 14215;; ^d^Department of Medicine, University at Buffalo, State University of New York, Buffalo, NY 14203;; ^e^Institute of Pharmacy, Pharmaceutical Microbiology, University of Greifswald, 17489 Greifswald, Germany;; ^f^Institute of Infection Medicine, Christian-Albrecht University of Kiel, 24105 Kiel, Germany;; ^g^Institute of Infection Medicine, University Medical Center Schleswig-Holstein, 24105 Kiel, Germany;; ^h^Friedrich Loeffler Institute of Medical Microbiology, University of Greifswald, 17475 Greifswald, Germany;; ^i^Central Unit for Infection Prevention and Control, University Medicine Greifswald, 17475 Greifswald, Germany;; ^j^Unita Operativa Complessa di Microbiologia e Virologia, Azienda Ospedaliera Universitaria Senese, I-53100 Siena, Italy;; ^k^Department of Microbiology and Immunology, University at Buffalo, State University of New York, Buffalo, NY 14203;; ^l^The Witebsky Center for Microbial Pathogenesis, University at Buffalo, State University of New York, Buffalo, NY 14203;; ^m^Centre d’Ingénierie des Protéines-InBioS, Université de Liège B-4000 Liège, Belgium

**Keywords:** antimicrobial resistance, bacterial pathogenesis, genomic epidemiology, nosocomial outbreak

## Abstract

A protracted outbreak of New Delhi metallo-β-lactamase (NDM)–producing carbapenem-resistant *Klebsiella pneumoniae* started in Tuscany, Italy, in November 2018 and continued in 2020 and through 2021. To understand the regional emergence and transmission dynamics over time, we collected and sequenced the genomes of 117 extensively drug-resistant, NDM-producing *K. pneumoniae* isolates cultured over a 20-mo period from 76 patients at several healthcare facilities in southeast Tuscany. All isolates belonged to high-risk clone ST-147 and were typically nonsusceptible to all first-line antibiotics. Albeit sporadic, resistances to colistin, tigecycline, and fosfomycin were also observed as a result of repeated, independent mutations. Genomic analysis revealed that ST-147 isolates circulating in Tuscany were monophyletic and highly genetically related (including a network of 42 patients from the same hospital and sharing nearly identical isolates), and shared a recent ancestor with clinical isolates from the Middle East. While the *bla*_NDM-1_ gene was carried by an IncFIB-type plasmid, our investigations revealed that the ST-147 lineage from Italy also acquired a hybrid IncFIB/IncHIB–type plasmid carrying the 16S methyltransferase *armA* gene as well as key virulence biomarkers often found in hypervirulent isolates. This plasmid shared extensive homologies with mosaic plasmids circulating globally including from ST-11 and ST-307 convergent lineages. Phenotypically, the carriage of this hybrid plasmid resulted in increased siderophore production but did not confer virulence to the level of an archetypical, hypervirulent *K. pneumoniae* in a subcutaneous model of infection with immunocompetent CD1 mice. Our findings highlight the importance of performing genomic surveillance to identify emerging threats.

## Significance

Carbapenem-resistant *Klebsiella pneumoniae* belongs to the “critical-priority” tier of bacterial pathogens as identified by the World Health Organization. Emerging “high-risk” lineages are responsible for difficult-to-treat, hospital-acquired infections and outbreaks around the globe. By integrating genomic and epidemiological data for isolates collected over 20 mo, this study revealed both the high, regional prevalence and the rapid spread, within a single hospital, of *K. pneumoniae* ST-147 in Italy. Besides resistance to nearly all antibiotics, this lineage carried a hybrid plasmid harboring a set of biomarker genes previously linked to hypervirulence. Convergence of resistance and virulence determinants is a major concern and these findings highlight the need for robust, global surveillance to monitor the emergence of high-risk *K. pneumoniae*.

*Klebsiella pneumoniae* is a leading cause of healthcare-associated infections including pneumonia, urinary tract, and bloodstream infections (1). Classical *K. pneumoniae* (cKp) frequently causes opportunistic infections in immunocompromised patients, the elderly, neonates, and patients with inserted medical devices (2). Of further concern, cKp strains can readily acquire antimicrobial resistances including extended-spectrum β-lactamases and carbapenemase-encoding genes (3). Prompting a major public health challenge, the global emergence and dissemination of multidrug-resistant *K. pneumoniae* (MDR-cKp) are attributed to a few successful clonal lineages, including newly identified “high-risk” sequence type (ST)-147 and ST-307 (4–6). Similar to ST-307, the MDR-cKp ST-147 lineage emerged in Europe during the mid-1990s, acquired plasmids encoding various carbapenemase genes (i.e., Verona Integron-Encoded metallo-β-lactamase [VIM] and New Delhi metallo-β-lactamase [NDM] as well as OXA-48 serine-carbapenemase variants) in the mid-2000s, and has now spread to all continents (4).

In recent years, a distinct hypervirulent pathotype (hvKp) has been identified, and is recognized clinically by invasive and disseminated infections, in otherwise healthy individuals, that include meningitis, liver abscesses, and endophthalmitis (2). However, the defining features of hypervirulence remain ambiguous. Phenotypically, hvKp isolates have been characterized primarily by their hypermucoviscosity, antimicrobial susceptibility, and greater production of siderophores. Genetically, several virulence genes, carried on large virulence plasmids and integrative conjugative elements (ICEs), encoding for the biosynthesis of siderophores (aerobactin [*iuc*], salmochelin [*iro*], and yersiniabactin [*ybt*]), the modulation of mucoviscosity and capsule synthesis (*rmpADC/rmpA2*), metabolite transporter *peg-344*, and the production of genotoxic polyketide colibactin (*clb*) have been linked to the hvKp pathotype (5, 7, 8).

Compounding the problem, convergent *K. pneumoniae* lineages with both virulence and resistance genes have been observed, albeit infrequently (3, 9, 10). Genotypic convergence most frequently occurs when MDR-cKp lineages acquire mobile genetic elements that carry the aforementioned virulence biomarker genes (11). Alarmingly, large hybrid plasmids that harbor both antimicrobial resistance and virulence genes have recently been reported in MDR isolates from multiple countries (6, 12–16). This includes sporadic instances of convergent *bla*_NDM_-carrying ST-147 isolates detected in the United Kingdom in 2018 and 2019 (13, 14). Yet, in most cases, the clinical impact and the resulting virulence potential of convergent lineages is not well-understood.

In this report, we investigated the emergence, genotypic convergence, phenotypic virulence, as well as the regional and nosocomial spread of an NDM-producing ST-147 clone causing an outbreak in Tuscany, Italy (17). This outbreak caused by an extensively drug-resistant (XDR) *K. pneumoniae* was first identified in November 2018 in the Tuscany region, where a significant increase in reported cases from seven hospitals led to expanded surveillance practices (18, 19). Recent surveillance data (20) suggest that, at the time of writing, this outbreak is ongoing with the risk of further transmission within and beyond Italy.

## Results

### Emergence of the NDM-1–Producing ST-147 Outbreak Clone in Italy.

**Fig. 1.**
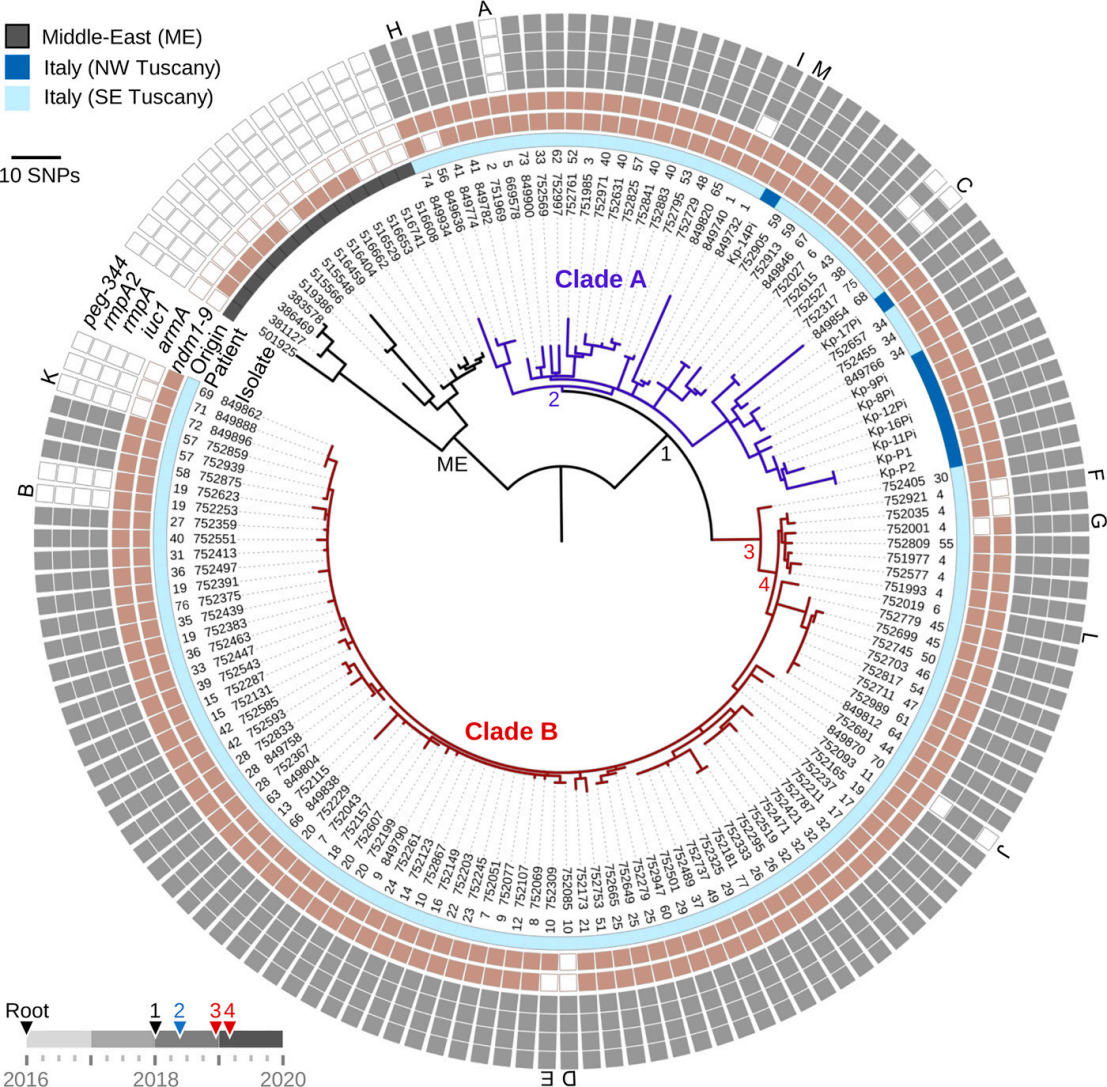
Maximum-likelihood core SNP-based phylogeny of *K. pneumoniae* ST-147 from Italy. Closely related isolates from the Middle East were included as an outgroup. Nodes of interest (all with bootstrap values >80) are labeled (ME, 1 to 4). When available, numerical patient identifiers are provided (1 to 77). Concentric rings indicate the presence (closed symbol) of a selection of plasmid-bound resistance and virulence genes. Variations in plasmid gene content detailed in *SI Appendix*, Fig. S3 are labeled (A to M). Predicted dates (Bayesian analysis is detailed in *SI Appendix*, Fig. S1) for the nodes of interest are indicated on the timeline (*Bottom Left*).

Between February 2019 and October 2020, a complete collection of NDM-producing *K. pneumoniae* ST-147 from the University Teaching Hospital of Siena and several long-term healthcare facilities in the southeast Tuscany region resulted in 117 isolates cultured from 76 patients (44% female; age range 27 to 96; median 75) (Dataset S1). Similar to ST-147 strains previously reported from the northwest (NW) region of Tuscany (19), these isolates were predominantly nonsusceptible to all tested penicillins, cephalosporins (including in combination with a β-lactamase inhibitor), carbapenems, and aminoglycosides (Dataset S2). These 117 isolates were mostly cultured from rectal swabs (74%, though the fraction of asymptomatic carriage is unknown) or urine (14%), respiratory tract (6%), or blood (5%) clinical samples. In 4/6 patients (ID nos. 20, 25, 26, and 40) with bloodstream infections, a positive rectal swab preceded blood cultures, suggesting gut colonization resulted in invasive disease (Dataset S1). Whole-genome sequencing (WGS) and phylogenetic analysis revealed that all ST-147 isolates from Italy [including nine publicly available genomes from bloodstream isolates collected in NW Tuscany (19)] were monophyletic (node 1) and showed high genetic relatedness (average pairwise distance from the nearest neighbor was 5.5 single-nucleotide polymorphisms [SNPs]) (Fig. 1). Bayesian analysis predicted their most recent common ancestor occurred in early 2018 (Fig. 1 and *SI Appendix*, Fig. S1), a few months before the first outbreak cases were reported from hospitals in Tuscany (17).

When compared with international ST-147 genomes (from both public databases and the Multidrug-Resistant Organism Repository and Surveillance Network collection), the outbreak clone from Italy most closely resembled (shortest distance was 45 SNPs) isolates collected from the Middle East between 2016 and 2017 (Fig. 1 and *SI Appendix*, Fig. S2). All isolates were predicted to be K antigen capsular biosynthesis loci K64 and O antigen–type O2 variant 1 (O2v1) and carried genes encoding the yersiniabactin siderophore system (*ybt9* harbored by a chromosomal ICEKp3) (Dataset S1). Further, the isolates from Italy carried a *bla*_NDM-1_ metallo-β-lactamase gene on an IncFIB-type plasmid (54,064-bp closed molecule, here named pSI0739-NDM) and a comparable plasmid (99% identity and 87% coverage) was found in 9 of 14 isolates from the Middle East.

### Detection of Hybrid Virulence and Resistance Plasmids.

Genomic comparisons further identified that, since divergence from the ancestor shared with the Middle East lineage, the ST-147 clone from Italy acquired a large hybrid IncFIB/IncHIB–type plasmid (335,489-bp closed molecule), subsequently referred to as pSI0739-ARMA-Vir. In addition to eight other antimicrobial resistance genes, pSI0739-ARMA-Vir carries the 16S ribosomal RNA methyltransferase *armA*, as well as virulence genes encoding a tellurium resistance operon (*terZABCDEF*), aerobactin biosynthesis (*iuc1*), putative hypermucoidy and capsule synthesis loci (*rmpADC*/*rmpA2*), and metabolite transporter *peg-344* (Fig. 1 and Dataset S3). When compared with public databases, both pSI0739-ARMA-Vir and pSI0739-NDM were nearly identical (*SI Appendix*, Fig. S3) to plasmids found in highly genetically related ST-147 isolates (*SI Appendix*, Fig. S2) reported from multiple UK hospitals in 2018 and 2019 (14).

Within the outbreak isolates from Tuscany, analysis of plasmid gene content was used to identify major excision events or plasmid loss (labeled A to M) (*SI Appendix*, Fig. S4). In five independent instances (A, B, I, J, and M) an ∼30- to 90-Kb segment containing some virulence genes was excised from pSI0739-ARMA-Vir (*SI Appendix*, Fig. S4 and Dataset S3). Insertion sequences (ISs) were often identified at the boundaries of the virulence island, suggesting transposition could play a role in the mosaic structure of these plasmids, as previously proposed (14). Notably, excision event B occurred within the host where (two out of five) serial isolates from patient 19 eventually lost all virulence genes (Fig. 1 and *SI Appendix*, Fig. S4). The *armA* gene cassette, flanked by IS*26* elements, was also excised on two separate occasions (E and F, the latter as the likely result of in-host evolution within serial isolates from patient 4). By contrast, the complete pSI0739-ARMA-Vir was lost in three of the later isolates (July and August 2020; event K), all highly genetically related but from distinct patients (Fig. 1 and *SI Appendix*, Fig. S4). Plasmid loss was also observed for pSI0739-NDM (three distinct events, D, G, and H) though, for sporadic cases, loss during subculturing cannot be excluded (*SI Appendix*, Fig. S4). As expected, susceptibility to the aminoglycosides and/or carbapenems was restored in these isolates (Dataset S2). Finally, while the backbone of pSI0739-NDM was lost (event L) in isolate 752019, *bla*_NDM-1_, carried on a Tn*125*-like transposon (21, 22), integrated into the canonical pSI0739-ARMA-Vir plasmid, resulting in the de novo formation of hybrid plasmid pSI0646A-ARMA-Vir-NDM. Importantly, near-identical hybrid plasmids carrying determinants for virulence, carbapenem (*bla*_NDM_), and broad-spectrum aminoglycoside resistances are now emerging in isolates from various countries in both ST-147 and distinct convergent *K. pneumoniae* lineages (*SI Appendix*, Fig. S3) (6, 14, 16, 22).

### ST-147 Outbreak Is Fueled by Nosocomial Spread.

Phylogenetic analysis of the isolates from Tuscany revealed the existence of two clades (differing by 32.9 SNPs on average), A and B, predicted to have emerged in mid-2018 and early 2019, respectively (Fig. 1 and *SI Appendix*, Fig. S1). Clade A was relatively heterogeneous (average pairwise distance from the nearest neighbor was 11.1 SNPs) and comprised 31 isolates from 23 patients at 3 distinct healthcare facilities in southeast Tuscany (Fig. 1 and Dataset S1). Additionally, clade A includes nine genomes from a hospital in NW Tuscany for which patient metadata are unknown (19). In contrast, clade B was highly homogeneous (average distance of 2.4 SNPs from the nearest neighbor) and grouped 86 isolates collected from 57 inpatients from the University Teaching Hospital of Siena.

**Fig. 2.**
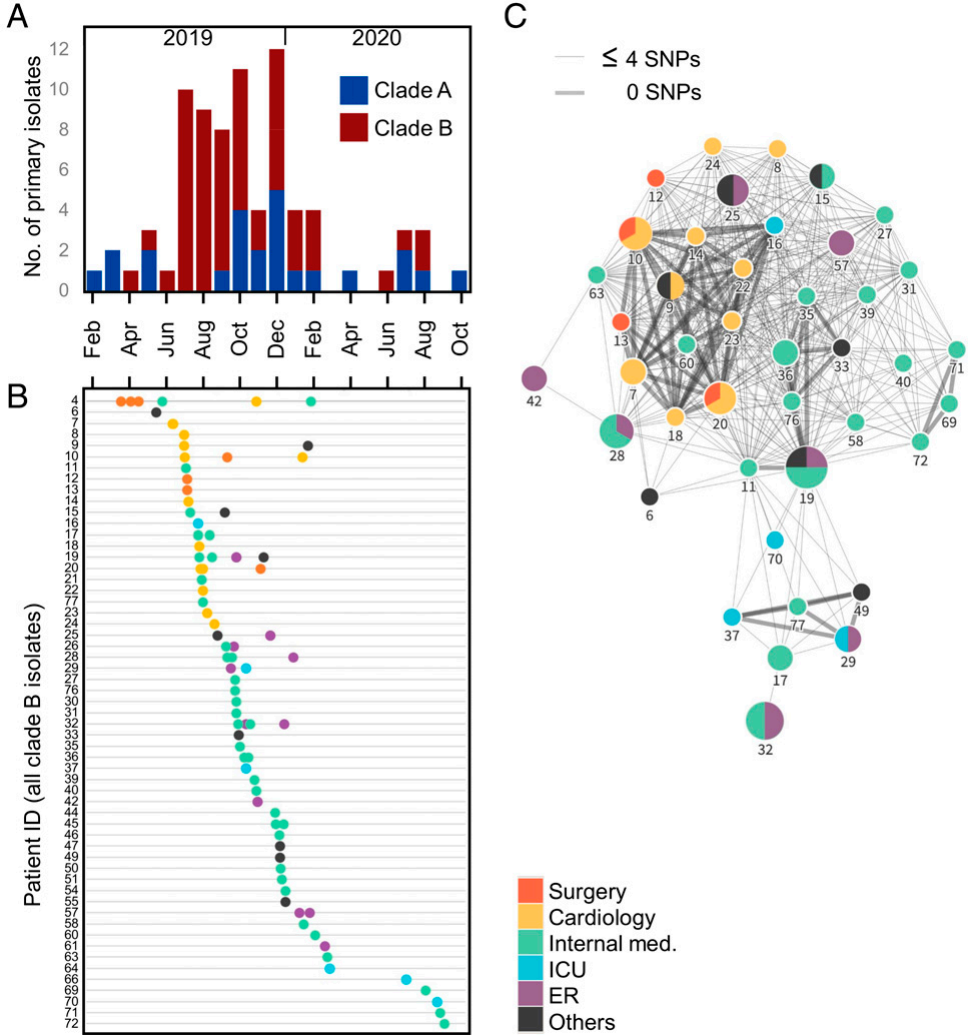
Rapid, nosocomial spread of clade B ST-147 clone. (*A*) Epidemic curve of all patients’ first isolate collected in southeast Tuscany and colored by clade. (*B*) Patient charts showing the date (*x* axis) and location (colored legend) of all first and serial clade B isolates (dots). (*C*) Network of patients with near-identical clade B isolates (≤4 SNPs). Nodes are labeled with their respective patient ID and indicate the location (colored pie chart) and number (size) of serial isolates.

Temporally, detection of the first clade A isolate from each patient (i.e., removing serial isolates) was relatively stable (1.2 new cases per month on average) across the 20-mo sampling period (Fig. 2*A*). By contrast, a large increase of new cases due to clade B isolates (7.1 cases per month) was observed over a 6-mo period, quickly following its emergence, from July to December 2019. Further, the majority (90%) of clade B isolates were collected from only five wards with few patients (*n* = 10) yielding positive, serial cultures from multiple wards (Fig. 2*B*). To trace possible nosocomial events, patient pairs that shared an isolate separated by four SNPs or fewer were identified and used to construct a network of possible transmission. This approach ultimately linked 74% (*n* = 42) of all patients represented in clade B and revealed that eight inpatients (ID nos. 7, 9, 10, 14, 18, 20, 22, and 23), all in the cardiology clinic in July and August of 2019, shared genetically identical isolates (Fig. 2 *B* and *C*).

While the last case detected in the cardiology clinic was in September 2019, this clone had seemingly spread throughout the hospital. Indeed, between September 2019 and October 2020, the internal medicine and emergency wards accounted for 75% of primary cases due to a clade B isolate (Fig. 2 *B* and *C*). Of note, the apparent reduction in isolates collected from patients between February and June 2020 was likely the result of hospital policies to reduce the number of nonurgent programmed hospitalizations in relation to the COVID-19 pandemic. New cases (e.g., patients 69 to 72) quickly reappeared from the same wards (suggesting a possible origin from contaminated surfaces) in late summer of 2020, indicating nosocomial transmission of clade B was not yet contained (Fig. 2 *A* and *B*).

### Convergent Evolution on Resistance and Virulence Determinants.

**Fig. 3.**
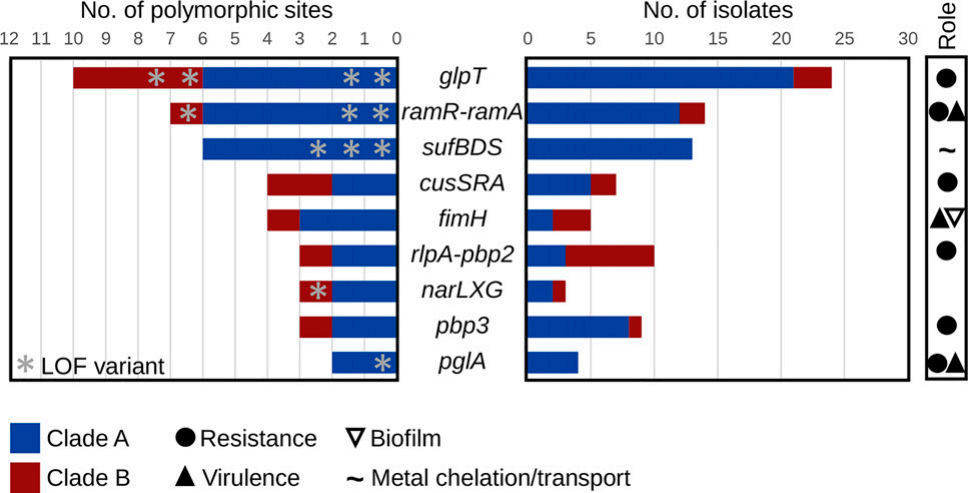
Convergent evolution on resistance and virulence genes. List of genes (or functional clusters of syntenic genes) that were repeatedly and independently (three or more) mutated among the outbreak isolates. Bars show the number of different mutations within each gene or functional cluster (*Left*) and the numbers of isolates with a mutated allele (*Right*). When available, and based on the literature, the role of genes and functional clusters in relevant phenotypes is indicated.

In addition to some isolates independently excising regions within or the whole pSI0739-ARMA-Vir and pSI0739-NDM plasmids, mutational convergence was also observed within specific genes and functional clusters. The most striking example was the gene *glpT* that encodes the main transporter responsible for the uptake of the fosfomycin antibiotic. Not accounting for synonymous (SYN) mutations, nine variants emerged independently in *glpT* (four predicted loss-of-function [LOF] and five nonsynonymous [NSY] mutations) and were found in 24 outbreak isolates, mostly from clade A (Fig. 3). With the exception of isolate 752737 (frameshift at the 3′ end of *glpT*), LOF mutations in *glpT* were associated with a large increase in fosfomycin minimum inhibitory concentrations (MICs), from 8 mg/L in the wild type to >64 mg/L in mutant isolates. Increased MICs (16 to >64 mg/L fosfomycin) were also observed in 14 of 18 isolates that carried an NSY mutation (Dataset S2). Four distinct NSY mutations in the *cusSRA* cluster, previously associated with copper, silver, and tigecycline resistance (23), were found in seven isolates though only small increases in MIC were generally observed in vitro (Dataset S2). In addition, the *ramR-ramA* gene cluster, consisting of the *romA-ramA* operon and the divergently transcribed *ramR* (resistance antibiotic multiple protein A) (24), was mutated at seven distinct sites (including two distinct LOF in *ramR* and one in *romA*) (Fig. 3). For *ramR*, two serial isolates from patient 4 with the same frameshift mutation demonstrated high-level tigecycline resistance (MIC ≥ 8 mg/L compared with ≤0.5 mg/L in the wild type) while an early stop codon resulted in decreased susceptibility (MIC = 2 mg/L) in isolate 752761 (*SI Appendix*, Fig. S5 and Dataset S2). Similarly, mutations in both *ramR* and *romA* were found in five genetically related isolates from NW Tuscany for which decreased susceptibility to tigecycline was previously noted (19) (Dataset S2). Further, Falcone et al. also reported that 1) isolates Kp-P1 and Kp-P2 carried *bla*_NDM-9_, which differs from *bla*_NDM-1_ by a single amino acid substitution and confers higher levels of resistance to carbapenems, and 2) isolates Kp-P1, Kp-P2, Kp-12Pi, and Kp-16Pi evolved resistance to colistin in part due to LOF mutations in *mgrB* (19). Here, isolate 849820 carried a truncated *mgrB* (Dataset S1) but remained susceptible to colistin (MIC < 0.25 mg/L). Convergent evolution was also observed for genes involved in cell-wall synthesis (*pbp2*, *pbp3*, *rlpA*) and glycosylation (*pglA*), adhesion to mucosal surfaces and virulence (*fimH*), nitrate respiration (*narLXG*), and iron binding and transport (*sufBDS*) (Fig. 3). The latter was recently reported as a potential factor underlying the persistence of an outbreak involving high-risk ST-258 *K. pneumoniae* (25).

### Fixation of Variants through Patient Transmission and within Host Evolution.

To further identify traits that possibly contributed to the success and spread of the ST-147 clone in hospitals in Tuscany, we identified variants that became fixed in the population at nodes of interest (excluding SYN and intergenic variants) (*SI Appendix*, Fig. S5*A*). A total of eight variants were shared in all isolates from Italy but missing in all Middle East genomes (node 1). Of particular interest were 1) a predicted LOF mutation in *cycA* which encodes a serine/alanine transporter that, when functional, allows the antibiotic d-cycloserine to cross the cell wall and reach its cytoplasmic target (26), 2) an NSY mutation in *pabA* involved in aromatic amino acid biosynthesis and previously characterized as a general infection requirement for *K. pneumoniae* (27), and 3) an NSY mutation in *hdfR* encoding for a transcriptional regulator involved in the complex regulation of capsule production and hypermucoviscosity (28).

Two NSY variants (in genes *fliY* and *ybiO*) arose in the last common ancestor at the root of clade A (node 2) while a total of nine variants arose at the root (nodes 3 and 4) of clade B (Fig. 1 and *SI Appendix*, Fig. S5*A*). Among them, variants in two genes could participate in the apparent success of clade B in spreading and persisting in the hospital: *metQ*, a d-methionine–binding lipoprotein, shown to be important for colonization (29), and *pal*, encoding a peptidoglycan-associated lipoprotein shown to provide protection against neutrophil phagocytosis and killing as well as resistance to gastrointestinal bile salts (30), a key ability for transmission via the fecal–oral route.

Similarly, longitudinal sampling of six serial isolates (March 2019 to January 2020) from patient 4 revealed a stepwise fixation of variants (*SI Appendix*, Fig. S5*B*). NSY mutations were present in genes involved in cell-wall biosynthesis/recycling (*rlpA*, *emtA*) and surface polysaccharide synthesis (*rffG*). The latter is part of a gene cluster that encodes the enterobacterial common antigen, a carbohydrate polymer thought to play a significant role in *K. pneumoniae* physiology and host interactions (27). Amino acid substitutions in the adhesin *fimH* and in the multifunctional regulator *mgrA* were also observed in these serial isolates.

### Pathogenicity of the Convergent ST-147 Clone from Italy.

**Fig. 4.**
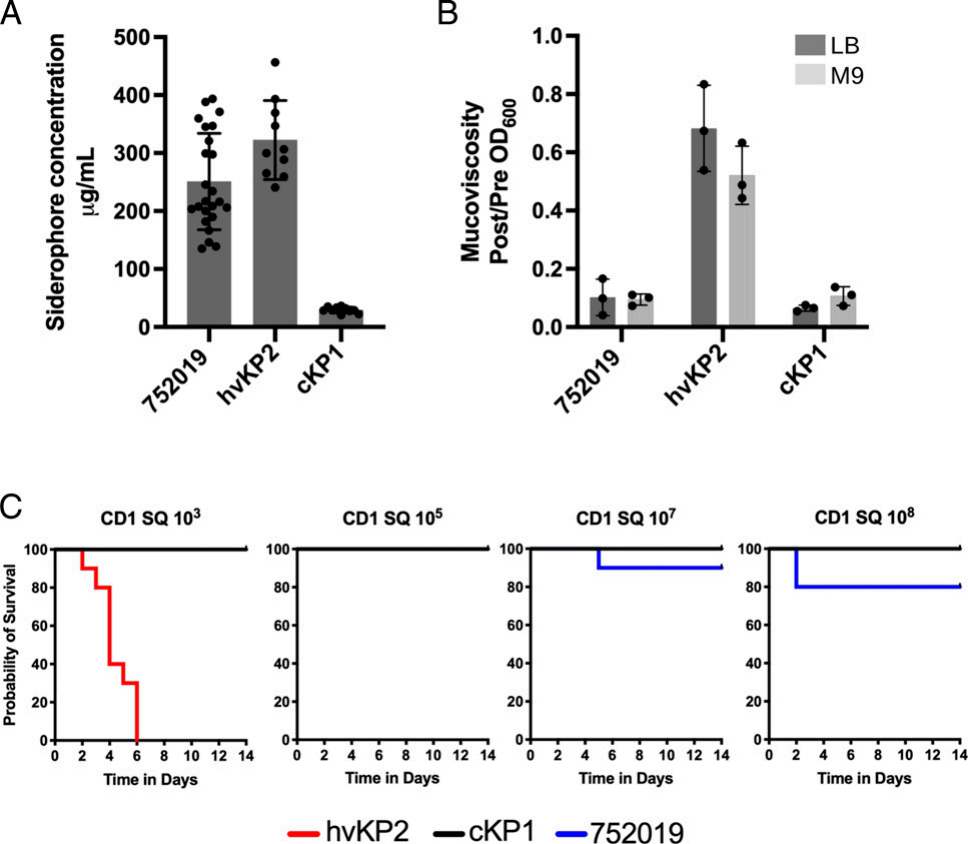
Pathogenicity of the convergent ST-147 clone from Italy. (*A*) Quantitative siderophore production of outbreak isolate 752019 carrying the hybrid pSI0646A-ARMA-Vir-NDM. Reference strains hvKP2 (hypervirulent pathotype) and cKP1 (classical pathotype) are shown throughout for comparison. (*B*) Mean mucoviscosity of outbreak isolate 752019 compared with reference *K. pneumoniae* isolates. For both in vitro assays, at least three independent biological replicates were performed for each isolate and all data points were reported as well as the mean ± SE. (*C*) Kaplan–Meier survival curves of outbred CD1 mice after SQ challenge with 10^3^, 10^5^, 10^7^, or 10^8^ CFU of outbreak isolate 752019 or reference isolates hvKP2 and cKP1. Total *n* = 10 (*n* = 5 in each of two independent experiments) for each titer for each strain.

While sequencing allowed for the detection of genotypic convergence, the virulence of this epidemic ST-147 clone remained to be established. Two distinct outbreak isolates (752019 and 752165) carrying IncFIB/IncHIB hybrid plasmids produced siderophore at levels well above the 30 µg/mL threshold predictive of the hvKp pathotype (∼230 µg/mL; Fig. 4*A* and *SI Appendix*, Fig. S6*A*) (31). The excision of virulence genes (including the *iuc* siderophore biosynthesis operon) in outbreak isolate 752253 (event B; *SI Appendix*, Fig. S4) resulted in siderophore levels comparable to the cKP1 control (*SI Appendix*, Fig. S6*A*). However, despite carrying the virulence genes *rmpADC* and *rmpA2*, isolates 752019 and 752165 showed mucoviscosity levels comparable to the cKP1 control (Fig. 4*B* and *SI Appendix*, Fig. S6*B*). Moreover, a subcutaneous (SQ) model of infection with immunocompetent CD1 mice determined that 752019 and 752165 were nonlethal at a challenge inoculum of 10^3^ CFU (colony-forming unit), unlike canonical hypervirulent isolate hvKP2 (Fig. 4*C*). Further, no lethality was observed with a 10^5^-CFU inoculum of the ST-147 isolates and only SQ challenges with 10^7^ and 10^8^ CFU revealed a slight increase in lethality (nonsignificant for 752019 [Fig. 4*C*]; *P* = 0.029 for 752165 at 10^8^ CFU [*SI Appendix*, Fig. S7*A*]) when compared with the cKP1 control, which was consistently found to be nonlethal at a challenge inoculum up to 2.5 × 10^8^ CFU (32). Likewise, an SQ challenge with strain PBIO1953 (6), a convergent ST-307 *K. pneumoniae* outbreak clone carrying a hybrid plasmid with extensive similarity to pSI0646A-ARMA-Vir-NDM (*SI Appendix*, Fig. S3), resulted in a mortality rate of 20% at the highest inoculum (not reaching statistical significance) compared with the cKP1 control (*SI Appendix*, Fig. S7*B*).

## Discussion

We report a comprehensive analysis of an NDM-producing ST-147 *K. pneumoniae* outbreak clone circulating in Tuscany, Italy. Although the number of colonized/infected patients was significantly lower than in the NW region (18), our study demonstrates that this epidemic, XDR clone has now spread throughout multiple healthcare facilities in the southeast region of Tuscany. Phylogenetic analysis suggests that this clone possibly emerged in early 2018 from an ST-147 MDR-cKp lineage, previously shown to be endemic in the Middle East (4), by acquiring a hybrid resistance/virulence plasmid. Furthermore, we also confirm the early concern about cross-border spread (17), as highly genetically related isolates and/or near-identical plasmids (*SI Appendix*, Fig. S3) are detected in emerging, convergent *K. pneumoniae* from hospitals around the globe (6, 14, 16, 22, 33).

Signs of strong selective pressure were observed in genes associated with drug resistance. With the prototypical ST-147 outbreak isolate resisting all first-line agents (i.e., β-lactams, carbapenems, fluoroquinolones, and aminoglycosides) (Dataset S2), effective treatment relies on second-line antibiotics (e.g., colistin, tigecycline, and fosfomycin) as well as combination therapies (34). In this context, the repeated, independent emergence of variants associated with resistance to either fosfomycin (*glpT*), tigecycline (*ramR*), or colistin (*mgrB*) is particularly worrisome and raises the concern of the emergence of a pan-resistant ST-147 epidemic lineage.

While WGS allows for the detection of convergence events in *K. pneumoniae*, the definitive impact on pathogenicity often remains unclear (3, 32). Compared with this ST-147 clone from Italy, the acquisition of the same combination of virulence genes was reported in distinct lineages of *K. pneumoniae* ST-11 from China and Egypt (15, 16) and ST-307 from Germany (6). Comparable virulence was observed in a *Galleria* insect model at 10^6^ CFU of ST-11 strains with and without the virulence plasmid (15). Furthermore, using clinical cohort stratifying, no increased mortality was observed in patients infected with ST-11 isolates carrying the virulence plasmid (15). The recent ST-307 epidemic clone from Germany carried a near-identical hybrid plasmid (*SI Appendix*, Fig. S3) and similarly showed increased siderophore production directly tied to the presence of the virulence genes (6). Here, an SQ challenge of immunocompetent CD1 mice unequivocally demonstrates that isolates from both lineages (ST-147 from Italy and ST-307 from Germany) did not exhibit a maximal hypervirulent phenotype [where lethality is observed with <10^3^ CFU (32)] and were only slightly more lethal than a cKP, at the highest inoculum tested. Nonetheless, a small increase in pathogenic potential may be clinically significant, particularly in the healthcare setting in which patients are variably immunocompromised.

Based on the current knowledge of *K. pneumoniae* pathogenicity, the reason(s) for the minimal virulence of the convergent ST-147 and ST-307 outbreak clones remains unclear. While increased siderophore production was historically associated with hvKp isolates (35, 36), we have recently shown that this measurement only partially correlates with virulence differences in vivo (32). The same observation was made for hypermucoviscosity (32), which, despite the presence of the required *rmpADC*/*rmpA2* genes, was not observed in this ST-147 clone. Other possible reasons are the absence of other known virulence factors (e.g., salmochelin and colibactin), genes whose role in virulence has yet to be recognized, virulence gene polymorphism, and differences in capsule type. In this latter possibility, ST-147 isolates are K antigen type 64, unlike the highly serum-resistant K1 and K2 capsules often associated with hvKp isolates (3). Finally, tight regulation of virulence determinants could mitigate the potential biological cost of expressing the many genes on these large hybrid plasmids, a barrier previously proposed as a reason for the relative rarity of convergent strains (3).

The role played by this IncFIB/IncHIB hybrid plasmid, and the selective forces underlying its emergence in distinct MDR *K. pneumoniae* lineages, remains a central question. While the presence of antibiotic resistance genes undoubtedly confers a selective advantage (37), a hypothesis would be that acquisition of other genes carried by this IncFIB/IncHIB hybrid plasmid confers increased colonization or persistence abilities, traits not examined in our systemic infection model. This could bear clinical significance as high loads of *K. pneumoniae* in the gastrointestinal tract have been associated with elevated risk of bacteremia (38). In our collection, most ST-147 isolates were recovered from rectal swabs. Previous work did demonstrate that MDR-cKp colonized poorly in comparison with hvKP1, suggesting an advantage might exist for carrying the virulence plasmid in the gut (39). In fact, increased siderophore production, the one feature observed in ST-147 and a direct result of the presence of the hybrid plasmid, has already been proposed to facilitate gut colonization and spread of various pathogenic Enterobacterales (40, 41). Finally, a tellurium resistance operon (*terZABCDEF*), harbored by the IncFIB/IncHIB hybrid plasmid in both ST-147 and ST-307 outbreak clones, has recently been proposed as a microbiome-dependent fitness factor involved in gut colonization in a murine model (42).

In summary, we identified a convergent clone of ST-147 that shares a recent common ancestor with isolates collected in the Middle East and has been sporadically detected in multiple hospitals in the United Kingdom. While this outbreak clone was XDR, the maximal hypervirulent phenotype was not observed in immunocompetent hosts in vivo despite the presence of a hybrid resistance/virulence plasmid. Further studies are needed to fully characterize the role of these plasmids and the drivers of their emergence in distinct lineages of MDR-Kp around the globe. Close monitoring of global *K. pneumoniae* populations, as well as ongoing, local efforts to contain the dissemination of such high-risk clones, is critical to impede the emergence of this troublesome pathogen.

## Materials and Methods

### Collection of Bacterial Isolates.

The complete set of XDR *K. pneumoniae* isolates (Dataset S1) was collected from the University Teaching Hospital of Siena (Azienda Ospedaliera Universitaria Senese), Italy, a 700-bed healthcare facility providing microbiological analyses in the framework of a regional surveillance network for several other local healthcare facilities that are part of the SE district and long-term healthcare structures. Samples were collected with informed consent as part of routine diagnostic purposes, contained no human genetic material, and were deidentified before the initiation of this retrospective research study. Bacterial species identification and antimicrobial susceptibility testing (Dataset S2) were carried out as detailed in *SI Appendix*.

### Whole-Genome Sequencing.

Genomic DNA was extracted and sequenced via Illumina MiSeq or NextSeq benchtop sequencers as previously described (43). For isolates 752165 and 752019 (alternate names SI-0739 and SI-0646A, respectively), long read sequencing was carried out using a MinION sequencer (Oxford Nanopore Technologies) as detailed in *SI Appendix*. All genomes and circularized plasmids have been deposited in the National Center for Biotechnology Information under BioProject PRJNA725484.

### Bioinformatic Analysis.

Multilocus sequence typing (MLST), virulence locus, capsule (K), and lipopolysaccharide (O) loci were identified using Kleborate v2.0.1 (Dataset S1) (12). The *peg-344* gene was identified using a BLASTn search of draft genome assemblies (accession no. NC_005249). AMRFinderPlus v3.9.8 (44) and ARIBA v2.14.4 (45) were used to identify resistance alleles. Core genome MLST (cgMLST) analysis was performed with Ridom SeqSphere+.

We created a recombination-free, SNP phylogeny (based on 587 variant sites) for ST-147 including the 117 outbreak isolates, 9 previously published genomes (19), and 14 Multidrug-Resistant Organism Repository and Surveillance Network genomes from the Middle East and Germany (Dataset S1). The methodology used for SNP calling and construction of a maximum-likelihood tree is detailed in *SI Appendix* and a full list of variants is provided (Dataset S4).

To date, internal nodes of interest on the phylogeny and Bayesian phylogenetic inference were performed using BEAST 2 v2.6.5 (46) under a coalescent constant population model using tip dates and a mean clock rate constrained to 1.45 × 10^−6^ substitutions per site per year, based on a previously reported evolution rate for the ST-147 lineage (47). Run parameters are detailed in *SI Appendix*.

For the patient transmission network, a pairwise SNP matrix for clade B isolates was obtained using Snippy v4.4.5 and snp-dist v0.7.0 (https://github.com/tseemann/snp-dists). Distances ≤4 SNPs between isolates from distinct patients were identified. In cases of serial isolates with varying distances, only the lowest interpatient SNP distance was retained. The resulting network of patients was visualized using Flourish (https://flourish.studio/).

Complete plasmids, pSI0739-ARMA-Vir and pSI0739-NDM, from isolate SI-0739 were used as reference for comparison. For both plasmids, gene presence/absence (Dataset S3) was determined using Roary (48) and the draft genomes of all outbreak isolates. Single-copy core gene (>98% identity over the full length) alignments were used to generate a pairwise SNP distance matrix for each plasmid (*SI Appendix*, Fig. S4 and Dataset S3). Plasmids from public databases were mapped against reference pSI0739-ARMA-Vir, pSI0739-NDM, and pSI0646A-ARMA-Vir-NDM using EasyFig (https://github.com/mjsull/Easyfig).

### Mucoviscosity and Quantitative Siderophore Assays.

Mucoviscosity of selected isolates was measured (ratio of OD_600_ values post/pre centrifugation at 1,000 × *g* for 5 min) after 24 h of growth (37 °C and 275 rpm) in Luria-Bertani (LB) or c-M9-te minimal medium (M9), as described previously (32). To quantify siderophore production, the isolates of interest were grown overnight at 37 °C in iron-chelated M9 minimal media containing casamino acids (c-M9-CA) and culture supernatants were assessed using the chromeazurol S dye assay as described (36). For both in vitro assays, at least three independent biological replicates were performed on different days for each strain and all data points were reported as well as the mean ± SD.

### Mouse Subcutaneous Infection Model.

Animal studies were reviewed, approved, and carried out under the direction of a veterinarian in strict accordance with the standard guidelines, as detailed in *SI Appendix*. CD1 male mice, 4 to 6 wk old, were obtained from Charles River Laboratories, quarantined for 5 d before use, and challenged via an SQ injection with the isolates of interest (100 µL of bacterial suspension serially diluted to the required titers in 1× phosphate-buffered saline diluted and injected using a 0.5-mL insulin syringe), as previously described (32). The animals were closely monitored for 14 d after challenge for the development of the study end points, survival, or severe illness (in extremis state)/death, which was recorded as a dichotomous variable.

### Statistical Analyses.

Statistical analyses were performed using GraphPad Prism 8.0. A log-rank (Mantel–Cox) test was performed to analyze in vivo infection model data. A Mann–Whitney *U* test was used to compare siderophore concentrations between strains. Bonferroni posttests were used to account for multiple comparisons. A *P* value <0.05 was considered statistically significant.

## Supplementary Material

Supplementary File

Supplementary File

Supplementary File

Supplementary File

Supplementary File

## Data Availability

Genome sequence data reported in this paper have been deposited in the National Center for Biotechnology Information under BioProject PRJNA725484. All study data are included in the article and/or supporting information.
