# Supplementary material for: Anatomy of an extensively drug-resistant Klebsiella pneumoniae outbreak in Tuscany, Italy
Source: Proc Natl Acad Sci U S A. 2021 Nov 24;118(48):e2110227118. doi: 10.1073/pnas.2110227118 (PMC8640832; doi:10.1073/pnas.2110227118)
Supplement: Supplementary File [file pnas.2110227118.sapp.pdf]

Anatomy of an Extensively Drug Resistant *Klebsiella pneumoniae* Outbreak in Tuscany, Italy

SI Appendix – Martin et al.

Materials and Methods

Species Identification and Antibiotic Susceptibility Testing.

Bacterial species identification and antimicrobial susceptibility testing were carried out using the MALDI Biotyper (Bruker Daltonics GmbH, Bremen, Germany) and the Becton Dickinson Phoenix M50 (Eysins, Switzerland). Carbapenemase production was confirmed using the mSuperCARBA medium (ChromAgar, Paris, France). β-lactamase genes were identified using a PCR analysis (Cica Geneus ESBL Genotype Detection kit, Cica Geneus AmpC Genotype Detection kit and Cica Geneus Carbapenemase Genotype Detection kit 2, Kanto Chemical Co., Tokyo, Japan), as recommended by the manufacturer. Isolates were sent to the Multidrug Resistant Organism Repository and Surveillance Network (MRSN) for further phenotypic characterization and genome sequencing. Confirmatory antibiotic susceptibility testing (AST) was performed in the MRSN College of American Pathologists (CAP)-accredited clinical lab using the Vitek 2 (card GN AST 71 and GN ID; bioMérieux, NC, USA) (**Dataset S2**). In addition, minimum inhibitory concentrations (MICs) of colistin and fosfomycin were determined in triplicate using the broth microdilution method following Clinical and Laboratory Standards Institute guidelines.

Long-read Sequencing and Assemblies.

Basecalling was performed using Guppy (configuration r9.4.1_450bps_hac), filtered using Filtlong (https://github.com/rrwick/Filtlong) and hybrid assembly was performed using Unicycler (1).

Prediction of SNPs and Genome Phylogeny

SNP calling was performed with Snippy v.4.4.5 (https://github.com/tseemann/snippy) using error corrected [Pilon v1.23 (2)] and annotated [Prokka v1.14.6 (3)] draft assembly of 752019 as the reference (**Dataset S4**). The core SNP alignment was filtered for recombination using Gubbins v2.4.1 (4), which identified 587 variant sites. A maximum likelihood tree was inferred with RAxML-NG v1.0.1 (5) using GTR+G (50 parsimony, 50 random) and was midpoint rooted in iTOL v. 5.5 (6) for visualization with metadata.

Temporal Phylogenetic Analyses

To evaluate the strength of the temporal signal, TempEst v1.5.3 was utilized to visualize the relationship between root-to-tip genetic distances for samples with known collection dates (7). The bayesian phylogenetic inference was performed using BEAST2 v2.6.5 on a recombination free alignment, removing samples with uncertain collection dates, and accounting for constant sites with beast2_constsites (https://github.com/andersgs/beast2_constsites). The GTR substitution model was selected based on evaluation of all possible substitution models in bModelTest v1.2.1 (8). The population was not found to be measurably evolving using marginal likelihood values estimated from isochronous and heterochronous models by the Nested Sampling package v1.1.0 (9). For further analyses, tip dates were used and the mean clock rate was constrained to 1.45×10^{-6} substitutions/site/year. The strict clock rate model was chosen after determining tree architecture and node dating were similar under clock models with more free parameters. With these models of import chosen, BEAST2 was run under a coalescent constant population model with a Markov chain Monte Carlo length of 1×10^{8} sampling every 1×10^{4} steps. Analyses were repeated four times to confirm consistency between the obtained posterior distributions. Parameter estimates were computed using Tracer v1.7.1. Posterior trees were combined with LogCombiner and

summarized in TreeAnnotator after a 10% burn-in. The final MCC target tree was visualized in FigTree v1.4.4 (https://github.com/rambaut/figtree) and annotated using iTOL (6).

Mouse Subcutaneous (SQ) Infection Model.

Animal studies were reviewed approved by the Veterans Administration Institutional Animal Care Committee and the University at Buffalo-SUNY and were carried out in strict accordance with the recommendations in the guidelines delineated in the "NIH Guide for the Care and Use of Laboratory Animals"(revised 1985) and the "Ethics of Animal Experimentation Statement" (Canadian Council on Animal Care, July, 1980) as monitored by the Institutional Animal Care and Use Committee. All efforts were made to minimize suffering. Veterinary care for the animals was supplied by the staff of Veterans Administration Animal Facility under the direction of a fully licensed veterinarian. Signs that were monitored and which resulted in immediate euthanasia using methods consistent with the recommendations of the American Veterinary Medical Association Guidelines included hunched posture, ruffled fur, labored breathing, reluctance to move, photophobia, and dehydration.

Supplemental References

1. R. R. Wick, L. M. Judd, C. L. Gorrie, K. E. Holt, Unicycler: Resolving bacterial genome assemblies from short and long sequencing reads. PLoS Comput Biol 13, e1005595 (2017).

2. B. J. Walker, *et al.*, Pilon: An Integrated Tool for Comprehensive Microbial Variant Detection and Genome Assembly Improvement. *PLoS ONE* **9**, e112963 (2014).

3. T. Seemann, Prokka: rapid prokaryotic genome annotation. *Bioinformatics* **30**, 2068–2069 (2014).

4. N. J. Croucher, *et al.*, Rapid phylogenetic analysis of large samples of recombinant bacterial whole genome sequences using Gubbins. *Nucleic Acids Research* **43**, e15–e15 (2015).

5. A. M. Kozlov, D. Darriba, T. Flouri, B. Morel, A. Stamatakis, RAxML-NG: a fast, scalable and user-friendly tool for maximum likelihood phylogenetic inference. *Bioinformatics* **35**, 4453–4455 (2019).

6. I. Letunic, P. Bork, Interactive Tree Of Life (iTOL) v4: recent updates and new developments. *Nucleic Acids Research* **47**, W256–W259 (2019).

7. A. Rambaut, T.T. Lam, L. Max Carvalho, O.G. Pybus, Exploring the temporal structure of heterochronous sequences using TempEst (formerly Path-O-Gen). *Virus evolution* **2**, vew007 (2016).

8. R.R. Bouckaert, R. R., A.J. Drummond, A. bModelTest: Bayesian phylogenetic site model averaging and model comparison. *BMC evolutionary biology*, **17**, 1-11 (2017).

9. P.M. Russel, B.J. Brewer, S. Klaere, R.R. Bouckaert, Model selection and parameter inference in phylogenetics using nested sampling. *Systematic biology*, **68**, 219-233 (2019).

Dataset S1. Characteristics of all isolates used in this study.

Dataset S2. Antibiotic susceptibility profiles for a selection of atypical ST-147 isolates.

Dataset S3. Genetic characteristics of pSI0739-ARMA-Vir and pSI0739-NDM.

Dataset S4. List of predicted variants in ST-147 isolates used in this study.

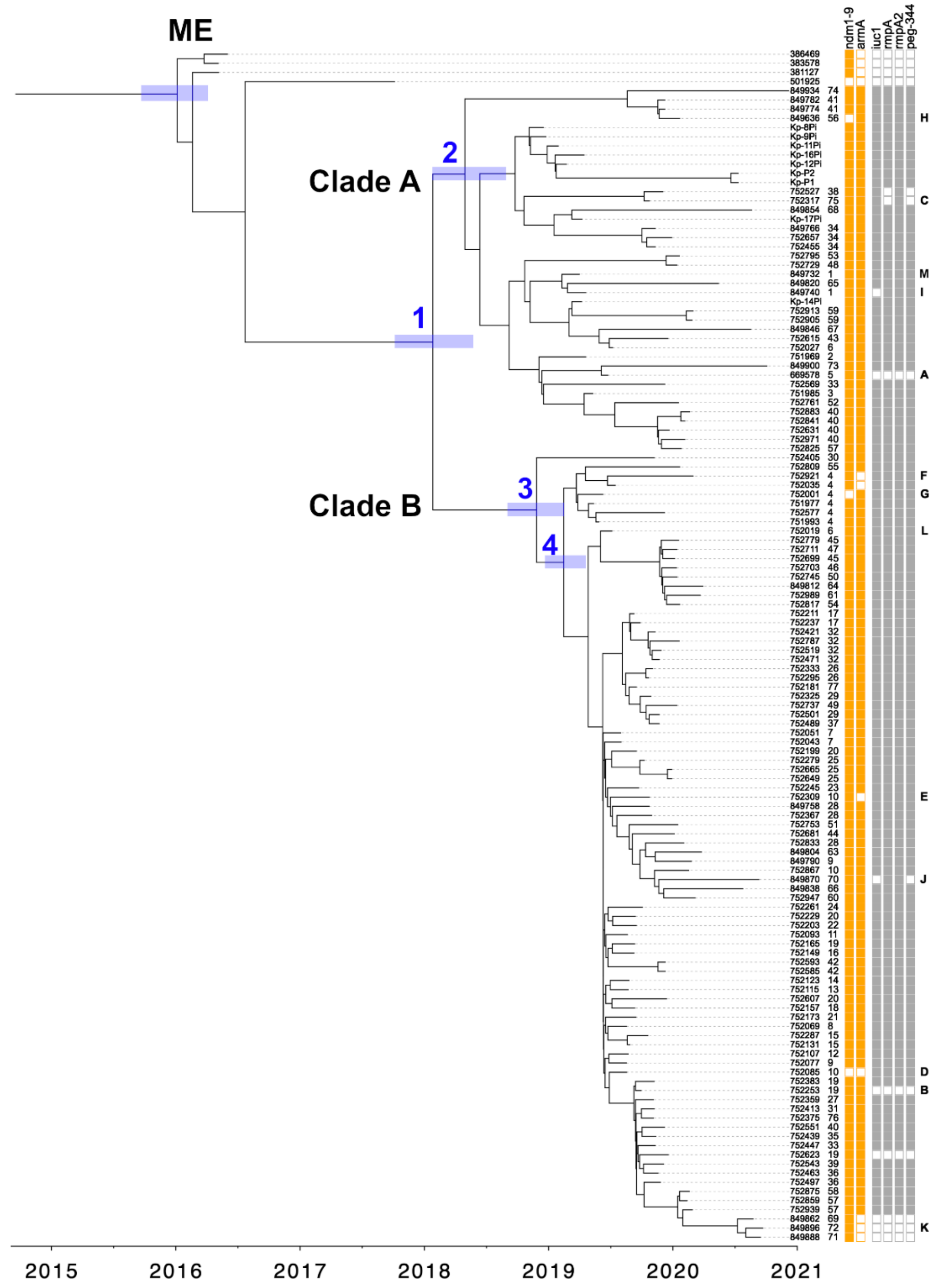

Figure S1. Bayesian tree inference of *K. pneumoniae* ST-147 from Italy. The 95% highest posterior density interval of node age estimates (ME, 1-4) are shown with transparent bars. When available, numerical patient identifiers are provided. Presence/absence matrix of a selection of plasmid-bound resistance and virulence genes are shown. Variations in plasmid content are labeled (A to M).

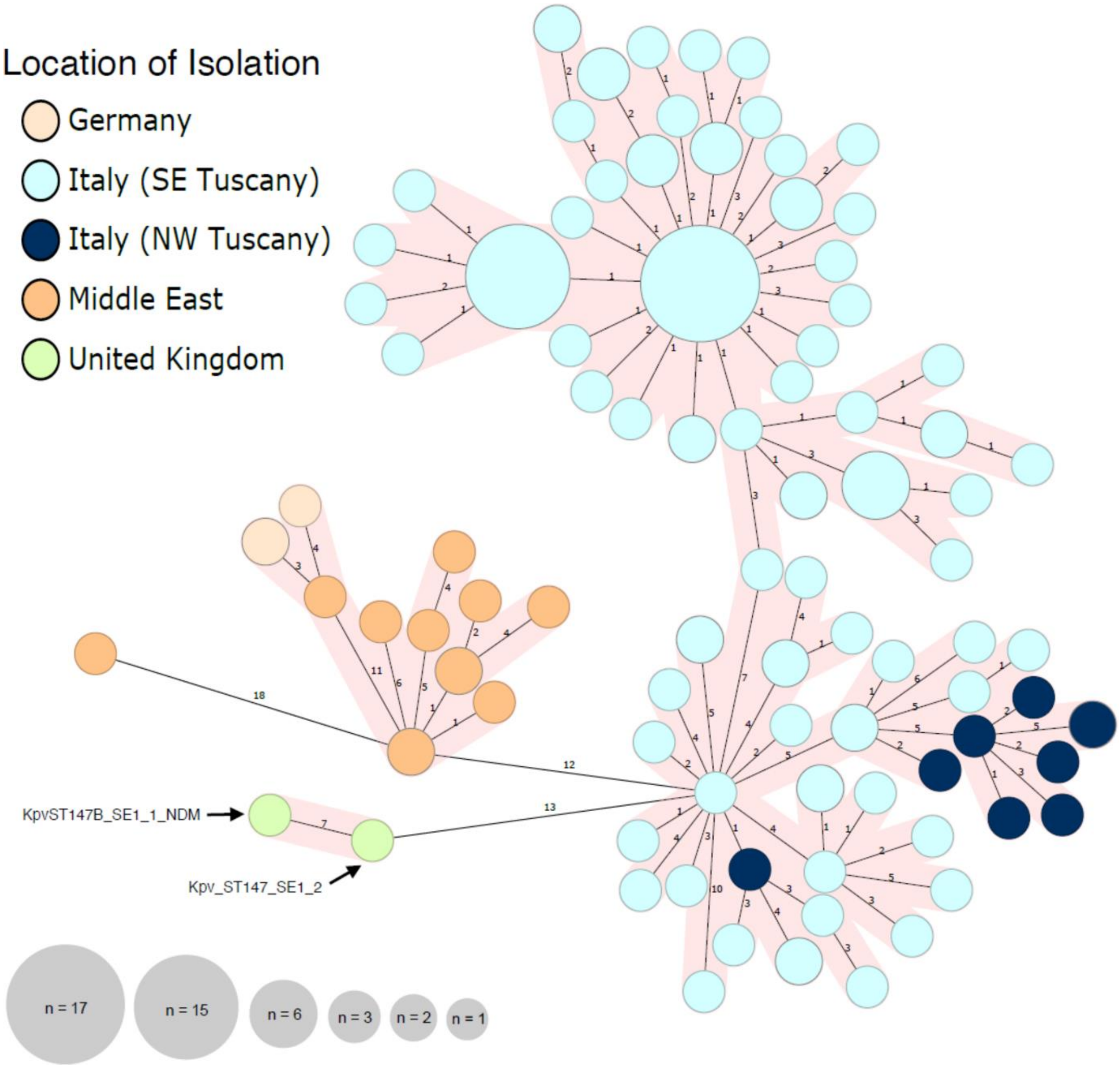

Figure S2. cgMLST minimum spanning tree of 143 ST-147 *K. pneumoniae* genomes used to determine the genetic relatedness of the Italy ST-147 outbreak clone to available ST-147 genomes from the MRSN collection and public databases. cgMLST allelic profiles are represented by circles, and circle sizes are proportional to the number of isolates sharing that profile. The line length connection the isolates represents the number of allelic differences between each profile. Isolates with 11 or less allelic differences are highlighted with pink shading around them.

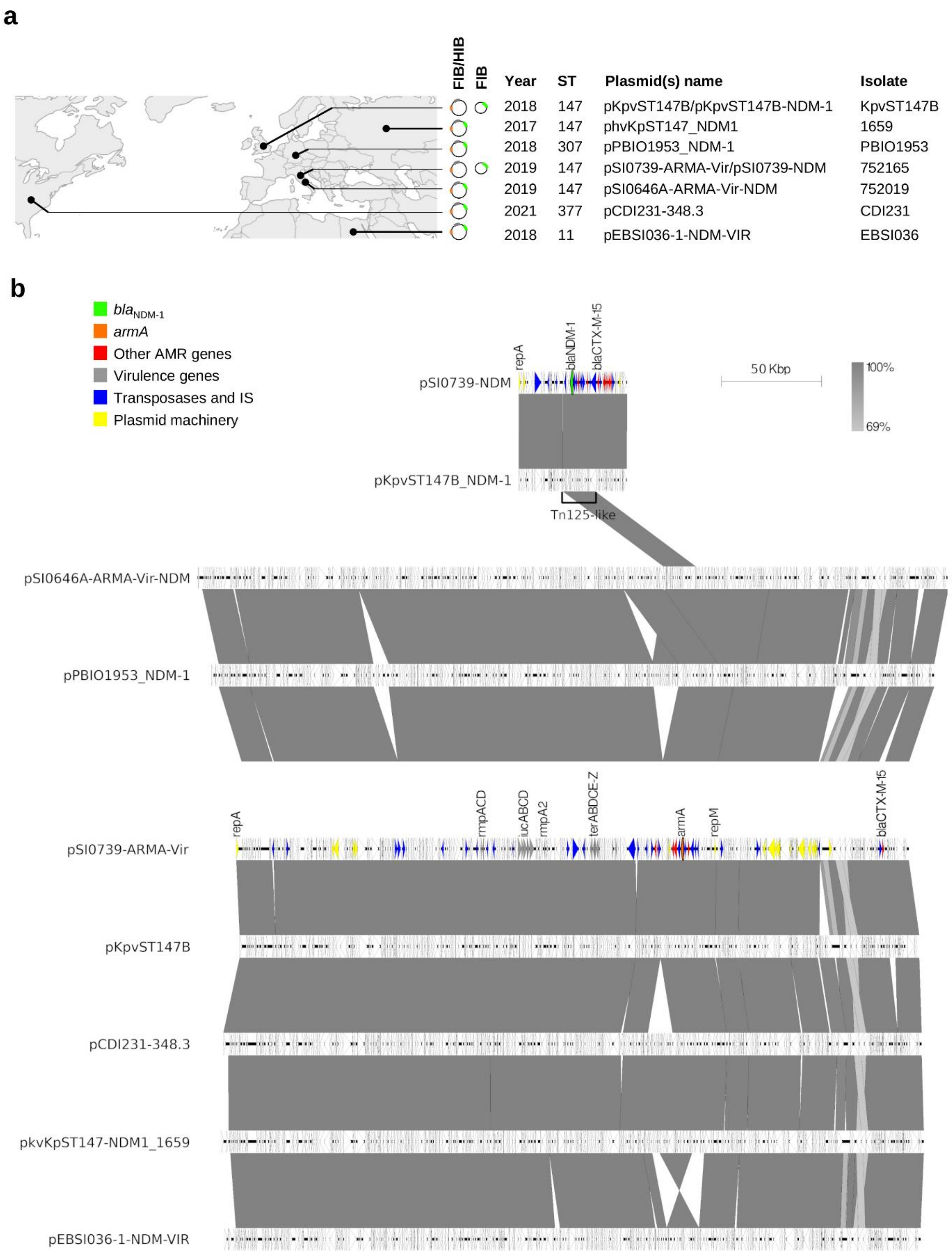
a
FIB/HIB
FIB
Year
ST
Plasmid(s) name
Isolate
2018 147 pKpvST147B/pKpvST147B-NDM-1 KpvST147B
2017 147 phvKpST147_NDM1 1659
2018 307 pPBIO1953_NDM-1 PBIO1953
2019 147 pSI0739-ARMA-Vir/pSI0739-NDM 752165
2019 147 pSI0646A-ARMA-Vir-NDM 752019
2021 377 pCDI231-348.3 CDI231
2018 11 pEBSI036-1-NDM-VIR EBSI036
b
bla NDM-1
armA
Other AMR genes
Virulence genes
Transposases and IS
Plasmid machinery
50 Kbp
100%
69%
repA
blaNDM-1
blaCTX-M-15
pSI0739-NDM
pKpvST147B_NDM-1
Tn125-like
pSI0646A-ARMA-Vir-NDM
pPBIO1953_NDM-1
repA
rmpACD
iucABCD
rmpA2
terABDCE-Z
armA
repM
blaCTX-M-15
pSI0739-ARMA-Vir
pKpvST147B
pCDI231-348.3
pkvKpST147-NDM1_1659
pEBSI036-1-NDM-VIR

Figure S3. Relatedness of pSI0739-NDM, pSI0739-ARMA-Vir, and pSI0646A-ARMA-Vir-NDM with a global collection of plasmids. (A) Highly related plasmids of interest were identified from public databases. IncFIB/IncHIB hybrid plasmids were found in distinct genetic lineages (ST-147, ST-307, ST-377, ST-11) from various countries (Italy, U.K., Russia, Germany, U.S., Egypt). In ST-147 isolates KpvST147B and 752165 (this study) *bla*$_{\text{NDM-1}}$ was located on IncFIB-type plasmids of ~54 kb. **(B)** Complete plasmids from public databases were mapped to closed plasmids pSI0739-NDM, pSI0739-ARMA-Vir, and pSI0646A-ARMA-Vir-NDM. The IncFIB-type plasmid pKpvST147B-NDM-1 is highly identical to pSI0739-NDM and both carried *bla*$_{\text{NDM-1}}$ located on a Tn*125*-like transposon. This *bla*$_{\text{NDM-1}}$ carrying transposon integrated into the canonical pSI0739-ARMA-Vir resulting in the *de novo* formation of plasmid pSI0646A-ARMA-Vir-NDM in isolate 752019. Homologous regions are shaded in dark grey.

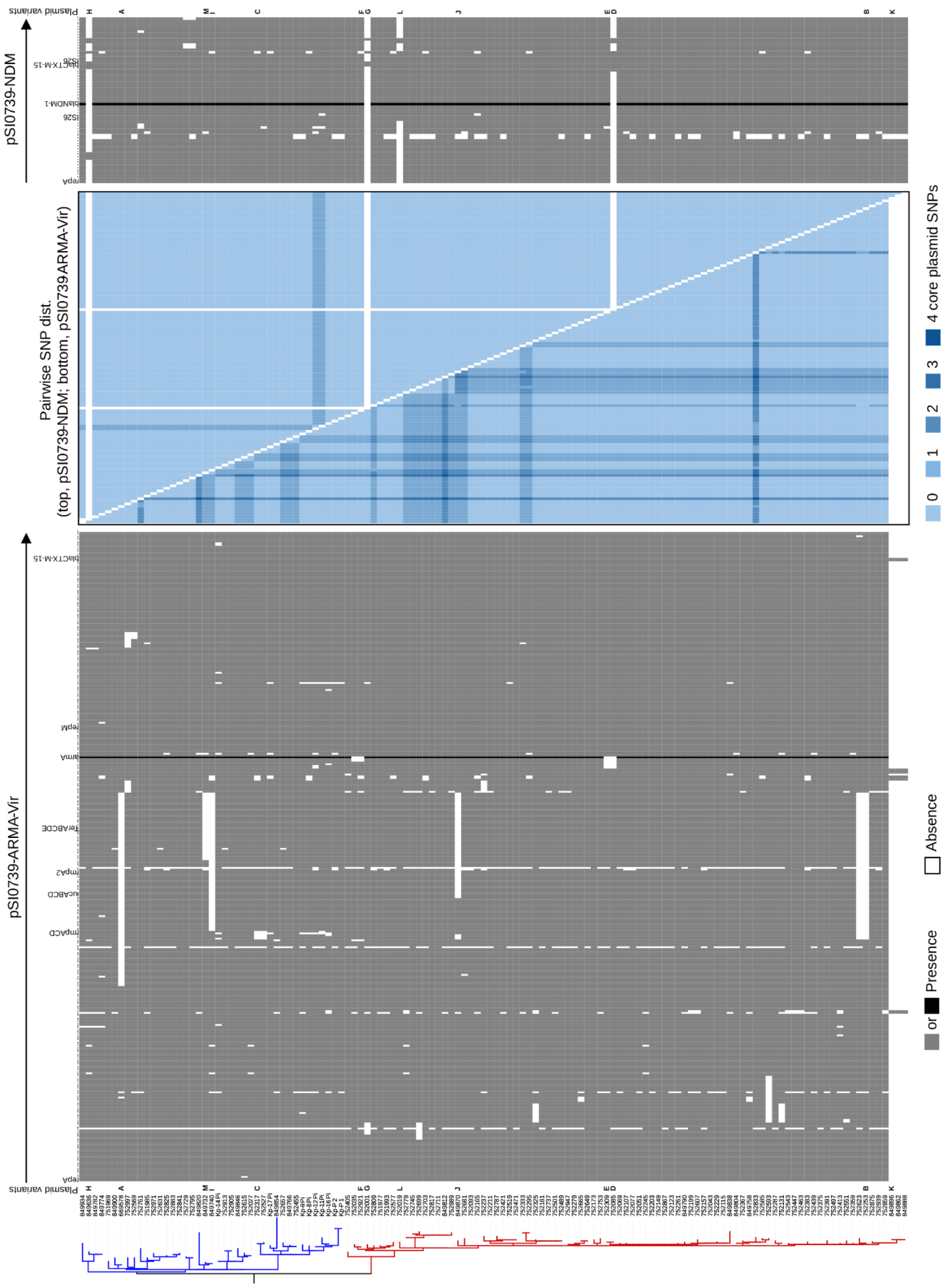
pSI0739-NDM
Pairwise SNP dist.
(top, pSI0739-NDM; bottom, pSI0739ARMA-Vir)
pSI0739-ARMA-Vir
Plasmid variants
0
1
2
3
4 core plasmid SNPs
or Presence
Absence

Figure S4. Plasmids comparisons across ST-147 outbreak isolates from Italy. For all outbreak isolates (ordered based on whole genome phylogeny), presence/absence of syntenic, sequential plasmid genes from pSI0739-ARMA-Vir (left) and pSI0739-NDM (far right) is indicated. Genes of interest are labelled, including $\mathit{bla}_{\mathrm{NDM-1}}$ and ArmA. Heatmap (middle) indicates the pairwise core-plasmid SNP distances for pSI0739-ARMA-Vir (below diagonal) and pSI0739-NDM (above diagonal). Missing values (*e.g.* comparison to self or lack of plasmid) are shown in white.

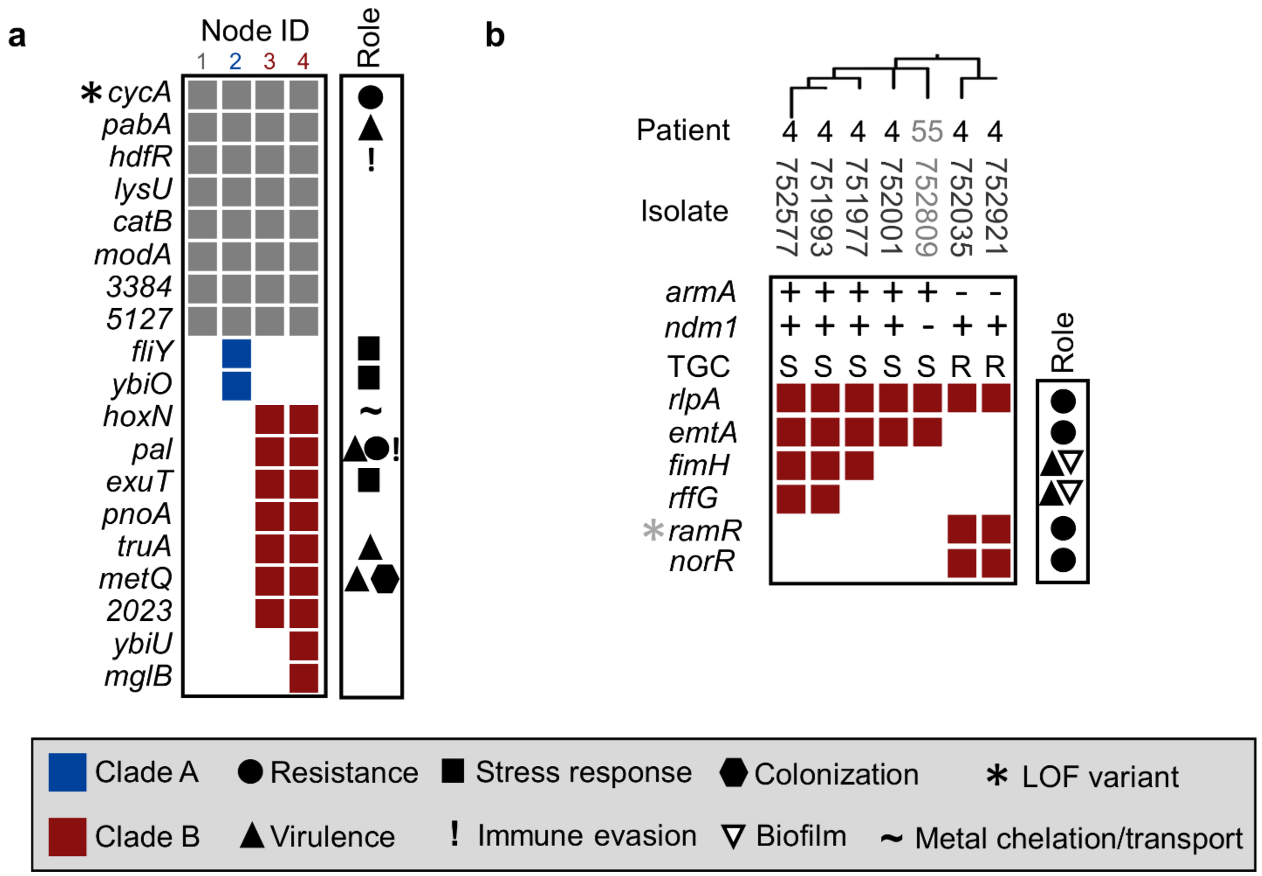

Figure S5. **(A)** Fixation of shared variants within the *K. pneumoniae* ST-147 outbreak strains (nodes 1-4 corresponding to Fig. 1). **(B)** Stepwise, fixation of variants found in 6 serial isolates from patient 4, clade B. For each gene, based on analysis of the literature, the role of its corresponding protein in resistance, virulence, metal transport, or biofilm formation is indicated.

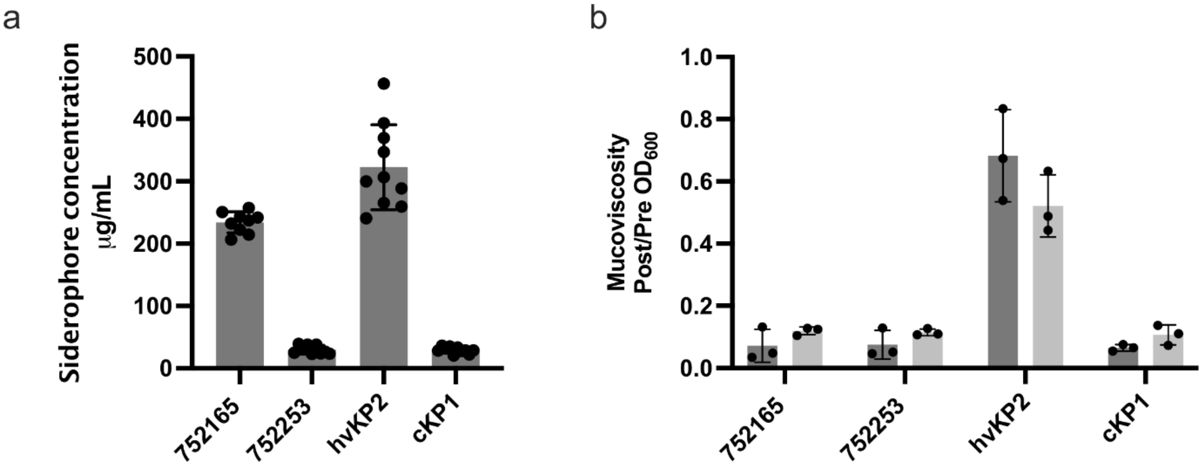

Figure S6. (A) Quantitative siderophore production of outbreak isolates 752165 (pSI0739-ARMA-Vir) and 752253 (pSI0739-ARMA-Vir, event B). **(B)** Mean mucoviscosity of outbreak isolates 752165 and 752253 compared to reference *K. pneumoniae* isolates. Independent assays were performed for both media (LB and M9). Reference hypervirulent (hvKP2) and “classical” (cKP1) isolates are shown throughout all panels for comparison.

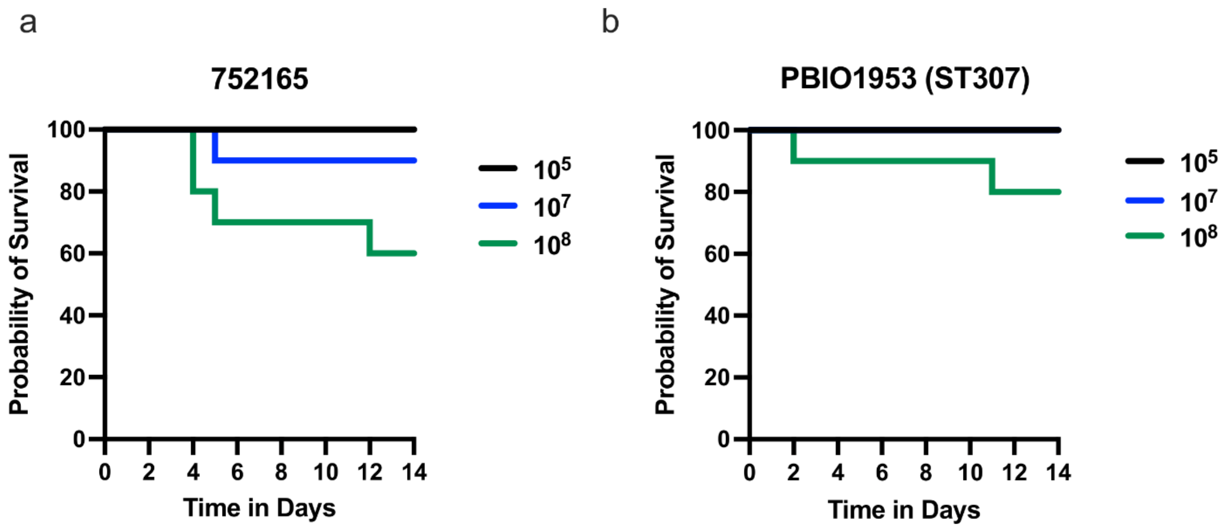

Figure. S7. Kaplan-Meier survival curves of outbred CD1 mice after subcutaneous (SQ) challenge with 10^5, 10^7, or 10^8 CFU of **(A)** outbreak isolate 752165 and (**B**) ST307 strain PBIO1953. Total *n*=10 (*n*=5 in each of 2 independent experiments) for each titer for each strain.
